# Effective operators in *t*-channel single top production and decay

**DOI:** 10.1140/epjc/s10052-018-6399-3

**Published:** 2018-11-12

**Authors:** M. de Beurs, E. Laenen, M. Vreeswijk, E. Vryonidou

**Affiliations:** 1 Nikhef, Science Park 105, Amsterdam, The Netherlands; 2 ITFA, University of Amsterdam, Science Park 904, Amsterdam, The Netherlands; 3 ITF, Utrecht University, Leuvenlaan 4, Utrecht, The Netherlands; 4 CERN Theory Division, 1211 Geneva 23, Switzerland

## Abstract

The production of a single top quark in the *t*-channel and its subsequent decay is studied at NLO accuracy in QCD, augmented with the relevant dimension-6 effective operators from the Standard Model Effective Theory. We examine various kinematic and angular distributions for proton-proton collisions at the LHC at 13 TeV, in order to assess the sensitivity to these operators, both with and without the top quark narrow width approximation. Our results will be helpful when devising strategies to establish bounds on their coefficients, including the amount of CP violation of the weak dipole operator.

## Introduction

Since its 1995 discovery [1, 2] by the CDF and D0 experiments the top quark has been an object of special interest in high-energy physics. Its large mass $m_t$, the largest of any known elementary particle, implies a strong coupling to the Higgs mechanism, and ensures that QCD corrections, proportional to $\alpha _s(m_t)$, are not overly large. The large mass also implies a large width, mainly composed of decays to a *W*-boson and a bottom quark, which prevents hadronisation, and enables clean transmission of spin information to the decay products. All of these characteristics invite careful testing in diligent comparisons of experiment and theory. The study of the single top production process has the added interest of directly involving the weak, charged-current interaction, allowing stringent testing of its flavour-changing, chiral nature.

**Fig. 1:**
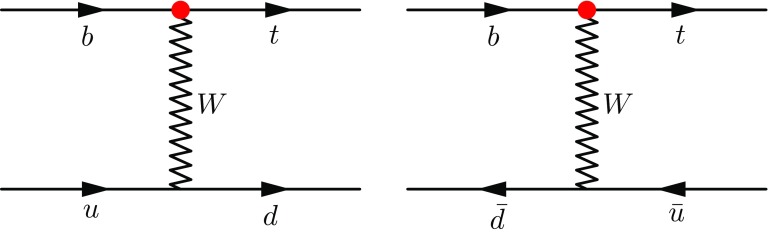
Feynman diagrams for *t*-channel single top production at LO. An incoming bottom quark and either an incoming up type (left) or anti-down type (right) quark exchange a virtual *W* boson, a so-called *t*-channel exchange. The outgoing *d* or ${\bar{u}}$ quark can be observed as a jet. The red vertex corresponds to Eq. 2.1, and allows, according to the SM, only left-handed top quarks to be produced

A key motivation behind such a precise scrutiny of the top quark is that its production and decay mechanisms should be especially sensitive to effects of physics beyond the Standard Model (SM). A systematic approach towards testing for the presence of such effects is the framework of effective field theory, in which the Standard Model is extended with higher-dimension operators that capture the effects of new physics in a model-independent way [3, 4]:

1.1 $$\begin{aligned} \mathcal{L}_{\mathrm {SM}} + \sum _i \frac{C_i }{\Lambda ^2} O^{[6]}_i +{\mathrm {hermitian\; conjugate}}, \end{aligned}$$

where $\Lambda$ is the scale of new physics, typically taken to be a few TeV, $O^{[6]}_i$ are dimension-6 operators, and $C_i$ their associated coefficient functions. If one assumes that these operators maintain SM symmetries one is lead to the SM Effective Field Theory (SMEFT) [5].

One of the virtues of single top production in the *t*-channel (for massless *b*-quarks) is that at leading order in QCD and at ${\mathcal {O}}(1/\Lambda ^2)$ only three operators $O^{[6]}_i$ with corresponding coefficients $C_i$ are required to parameterise new physics effects: $O^{(3)}_{\varphi Q}$, $O_{tW}$ and $O^{(3)}_{qQ,rs}$:

1.2 $$\begin{aligned} O_{\varphi Q}^{(3)}= & {} i \frac{1}{2} y_t^2 \left( \varphi ^\dagger \overleftrightarrow {D}^I_\mu \varphi \right) \left( {\bar{Q}}\gamma ^\mu \tau ^I Q\right) \end{aligned}$$

1.3 $$\begin{aligned} O_{tW}= & {} y_t g_w\left( {\bar{Q}}\sigma ^{\mu \nu }\tau ^It\right) {\tilde{\varphi }}W_{\mu \nu }^I \end{aligned}$$

1.4 $$\begin{aligned} O^{(3)}_{qQ,rs}= & {} \left( {{\bar{q}}}_r\gamma ^\mu \tau ^I q_s\right) \left( {{\bar{Q}}}\gamma _\mu \tau ^I Q\right) , \end{aligned}$$

where we have followed the notation and normalisation choice given in [6], and dropped the superscript denoting that the operators are of dimension-6. These operators run and mix under renormalisation group evolution [7–9], but we shall omit these effects in our analysis.

Let us note here that more operators can contribute starting at ${\mathcal {O}}(1/\Lambda ^4)$ such as the operators involving right handed bottom quarks, e.g. the dipole operator of the bottom quark, whose contributions are suppressed by the bottom mass at ${\mathcal {O}}(1/\Lambda ^2)$. Four-fermion operators involving right-handed light quarks can also be relevant at ${\mathcal {O}}(1/\Lambda ^4)$ [10], but these are eliminated if one assumes Minimal Flavour Violation [11]. In general we assume the contribution from dimension-8 operators to be sufficiently suppressed by their associated $1/\Lambda ^4$ prefactor. We shall however use order $1/\Lambda ^4$ contributions to the cross section arising from squared contributions of dimension-6 ones to assess uncertainties. Finally flavour changing interactions can also contribute to single top production, but we do not consider this here. For a recent global analysis of top-quark related flavour changing interactions in the effective operator framework see [12]. Global SMEFT constraints in the top sector are obtained in [13, 14].

This paper assesses the effect of the limited set of dimension-6 operators on single top quark production in the *t*-channel (for brevity we show results for top production, but the same observations can be made in anti-top production). We do so moreover at next-to-leading order (NLO) in QCD, including top quark decay to *W* and *b*, both in the narrow top width approximation (NWA), and by producing the *Wb* directly, including non-resonant contributions.

The paper is structured as follows. In the next section we discuss the necessary background to single top production in SMEFT. In Sect. 3 we present our results, highlighting the opportunities in constraining the dimension-6 operators with present and future LHC data. In the final section we present our conclusions.

## Single top production in SM extended to dimension-6

To establish our context we recall here some basic aspects of single top production, and the associated charged current interaction. The leading order diagrams for *t*-channel single top production are shown in Fig. 1.

The essence of the single top production channel in the SM is that the top quark is produced through an interaction with a *W* boson. This interaction corresponds to the following term in the SM Lagrangian

2.1 $$\begin{aligned} \mathcal{L}^{\mathrm {SM}}_{Wtb} = -\sum _{f=d,s,b}^3\frac{gV_{tf}}{\sqrt{2}}\, {\bar{q}}_f(x) \gamma ^\mu P_L t(x)\,W_{\mu }(x) + {\mathrm {h.\;c.}},\nonumber \\ \end{aligned}$$

The coupling strength is denoted by *g*, and top quark *t*(*x*) and *W*-boson $W_\mu (x)$ fields are indicated, as are quark fields $q_f(x)$, where $f=d,s,b$ indicates down, strange or bottom quarks. The coefficient $V_{tf}$ is an element of the Cabibbo–Kobayashi–Maskawa (CKM) matrix. Also shown is the projection operator $P_L$ which projects onto the left-handed (V-A) part of the top quark. Once produced, the top (or anti-top) quark decays almost always into a *b* quark and a *W* boson which subsequently decays to a positron (or electron) and the corresponding (anti-)neutrino.^1^ The notable aspect of this decay is that there is a near-perfect correlation between the flight direction of the positron in the top quark rest frame, and the top quark spin [15, 16]. As the positron is easily detected, this correlation allows a direct determination of the top quark spin, and the handedness of the coupling from the positron angular distribution.

Any new physics altering the *Wtb* interaction can then be probed by studying single top production and decay. The SMEFT can parameterise deviations from the SM predictions and can be used to make quantitative predictions to be compared with experimental data.

The operators of Eqs. (1.2)–(1.3) modify the *Wtb* interaction in the following way

2.2 $$\begin{aligned} \mathcal{L}^{\mathrm {dim-6}}_{Wtb}= & {} -\frac{g}{\sqrt{2}} {\bar{b}}(x) \gamma ^\mu P_L t(x)\,W_{\mu }(x) \left( 1+ \frac{C^{(3)}_{\varphi Q} y_t^2 v^2}{2\Lambda ^2} \right) \nonumber \\&+ \frac{2 \,g \,v \,y_t \, C_{tW}}{\Lambda ^2} {\bar{b}}(x) \sigma ^{\mu \nu } P_R t(x)\,\partial _{\nu } W_{\mu }(x) + {\mathrm {h.\; c.}},\nonumber \\ \end{aligned}$$

where $v = 246$ GeV is the Higgs doublet vacuum expectation value, and $y_t$ the top quark Yukawa coupling. Here and below we assume $V_{tb}=1$. Note that the four-fermion operator of Eq. (1.4) introduces a contact *udtb* interaction. The impact of these operators can be already seen by considering the partonic single top cross section, which at ${\mathcal {O}}(1/\Lambda ^2)$ can be written schematically as

2.3 $$\begin{aligned} \frac{d\sigma _{ub\rightarrow dt}}{d \cos \theta }= & {} \left( 1 + \frac{C^{(3)}_{\varphi Q } y_t^2 v^2}{\Lambda ^2} \right) k_1( \theta ) + \frac{C^{(3)}_{qQ,rs}}{\Lambda ^2} k_2(\theta )\nonumber \\&+\frac{\mathrm {Re\,}C_{tW}}{\Lambda ^2} k_3(\theta ), \end{aligned}$$

where the $k_i$ are known functions of $\theta$, the angle between the incoming bottom quark direction and the top quark flight direction in the partonic center-of-mass frame.

An interesting feature of this production cross section is that each of the coefficients $C^{(3)}_{\varphi Q}$, $C^{(3)}_{qQ,rs}$ and $\mathrm {Re\,} C_{tW}$ is associated with a specific angular dependence, enabling one to determine, or at least bound, the individual contributions experimentally.

**Fig. 2:**
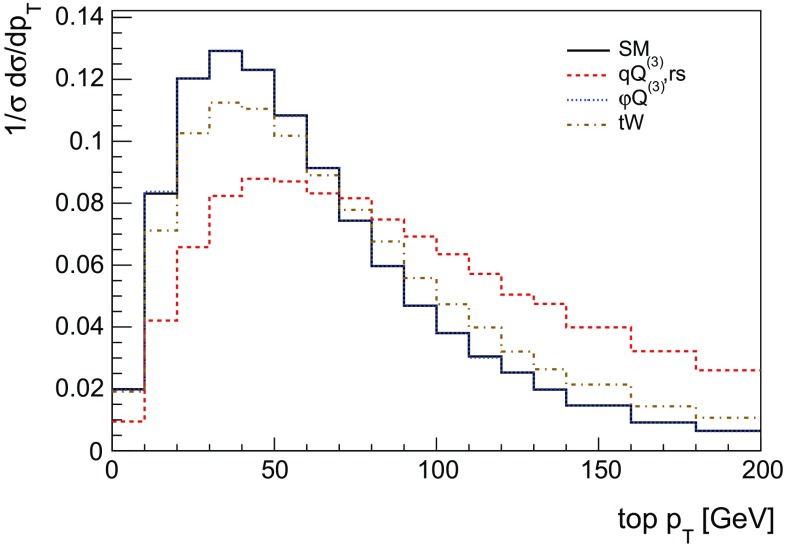
The normalised leading order parton-level differential cross section as a function of the transverse momentum of the top quark. The expectation of the SM together with the interference effects of the three effective operators of interest are shown

The operator $O^{(3)}_{\varphi Q}$ only modifies the magnitude of the *Wtb* interaction as shown in Eq. (2.2), but does not change the angular dependence of the SM prediction. By contrast, the operator $O^{(3)}_{qQ,rs}$, with corresponding real coefficient $C^{(3)}_{qQ,rs}$, represents a four-quark contact interaction and noticeably affects the angular distribution of the top quark production angle. Of course, Eq. (2.2) addresses only the dominant, lowest order parton process $u+b\rightarrow d+t$. Other partonic processes also contribute but the different angular behaviour of the partonic cross section predicted by the different operators directly translates into different shapes of the top transverse momentum distribution. This is illustrated in Fig. 2, where the effect of $C^{(3)}_{qQ,rs}$ on the top $p_T$ distribution is clearly distinguishable. Finally, the contribution of $\mathrm {Re\,}C_{tW}$ has a signature again different from the other two operators, but its effect is smaller and is better determined in the decay of top quarks than in their production [6].

The above discussion is somewhat simplified as it refers to the lowest order contributions in both QCD and the EFT expansion. Next-to-leading order (NLO) QCD corrections can be also relevant and can potentially modify the relative contributions of the operators. At NLO in QCD, the chromomagnetic dipole operator, $O_{tG}$, contributes as discussed in [6] whilst additional operators contribute at ${\mathcal {O}}(1/\Lambda ^4)$. We omit these operator contributions in this work but in future work we intend to take them into consideration.^2^

Given the different angular distributions already observed at the level of the partonic cross section, it is interesting to fully explore *t*-channel single top production in the presence of the dimension-6 operators and provide the relevant predictions for the LHC. In the following sections we will therefore study single top production in the presence of the dimension-6 operators in Eqs. (1.2–1.4) both at the inclusive and differential level, as well as including top decays, and we will identify observables that can be used to bound the values of the corresponding coefficients $C^{(3)}_{\varphi Q}$, $C^{(3)}_{qQ,rs}$ and $C_{tW}$.

## Numerical studies

To study the impact of the three operators on single top production we compute the corresponding contributions at LO and NLO matched to the parton shower (PS). The computation is performed within the MadGraph5_aMC@NLO (MG5_aMC) framework [22], and uses the NLO EFT implementation of Ref. [6]. While [6] produces results for stable top quarks, we will also consider the top quark decays. This can by achieved by either decaying the top-quark in MadSpin [23] or by following the procedure of resonance-aware PS matching presented in [24], to produce a *Wbj* final state. By decaying the *W* boson in MadSpin, we retain spin information. Our setup is fully differential and allows us to assess the impact of NLO corrections as well as the impact of the operators entering either in the production or in the decay, or both, for any observable.

To facilitate discussion we first fix our notation. Assuming one insertion of each operator, we can write the matrix element for single top production in the form

3.1 $$\begin{aligned} {\mathcal {M}} = {\mathcal {M}}_{\text {SM}} + \sum _i\frac{1\mathrm{TeV}^2}{\Lambda ^2}C_i \,{\mathcal {M}}_i\,, \end{aligned}$$

where the ${\mathcal {M}}_i$ are defined as having precisely one insertion of operator $O_i$ in all possible ways. We have normalised the new physics scale $\Lambda$ in units of TeV. In physical observables, such as the production cross section and the top width, the matrix element enters squared. The squared amplitude takes the form:

3.2 $$\begin{aligned} |{\mathcal {M}}|^2= & {} \mathcal {|M|}_{\text {SM}}^2 + \sum _i\frac{1\mathrm{TeV}^2}{\Lambda ^2}C_i \, 2\mathrm {Re}\left( {\mathcal {M}}^{*}_{\text {SM}} \, {\mathcal {M}}_i \right) \nonumber \\&+ \sum _{i\le j} \frac{1\mathrm{TeV}^4}{\Lambda ^4}C_iC_j \, \mathcal {|M|}^2_{i,j}, \end{aligned}$$

assuming for simplicity real operator coefficients. From here onwards the contribution to the cross section from the interference term with the SM ($\propto 2\mathrm {Re}\left( {\mathcal {M}}^{*}_{\text {SM}} \, {\mathcal {M}}_i \right)$) will be denoted by $\sigma _i$, while the additional squared terms ($\propto \mathcal {|M|}^2_{i,j}$) will be denoted by $\sigma _{i,j}$. In this notation, the cross section can be parameterised as:

3.3 $$\begin{aligned} \sigma =\sigma _{\text {SM}}+\sum _i\frac{1\mathrm{TeV}^2}{\Lambda ^2}C_i \, \sigma _i +\sum _{i\le j} \frac{1\mathrm{TeV}^4}{\Lambda ^4}C_iC_j \, \sigma _{i,j}. \end{aligned}$$

We will present results for all three terms. We recall here our remark in Sect. 1 that the $\mathcal{O}(1/\Lambda ^4)$ terms represented by the $\sigma _{i,j}$ are far from complete, and we use them here only to estimate uncertainties in the EFT expansion.

**Table 1:** Contributions to the cross section in pb for *t*-channel single top production at 13 TeV, as parameterised in Eq. (3.3). These values have been extracted from fitting Eq. (3.3), to a hundred computed cross sections with randomly chosen coupling strengths for the effective operators, both for LO and NLO separately. The statistical errors for each contribution in the table is below 1% except for the $\sigma _{qQij,tW}$ term at NLO, which is at 1.1%. The right-hand-side column shows the *K*-factor, which is defined for each row as the ratio of the NLO over the LO prediction. By the subscripts *tW* and *itW* we denote the contributions of the real and imaginary parts of $C_{tW}$ respectively

Operator	LO		NLO		*K*
	$\sigma$ [pb]	$\frac{\sigma }{\sigma _{\text {SM}}}$ [%]	$\sigma$ [pb]	$\frac{\sigma }{\sigma _{\text {SM}}}$ [%]	
$\sigma _{\text {SM}}$	123	–	137	–	1.12
$\sigma _{qQ,rs^{(3)}}$	$-$ 92.3	$-$ 75.3	$-$102	$-$ 74.7	1.11
$\sigma _{\varphi Q^{(3)}}$	14.6	11.9	16.3	11.9	1.12
$\sigma _{tW}$	3.05	2.49	3.57	2.6	1.17
$\sigma _{itW}$	–	–	–	-	–
$\sigma _{qQ,rs^{(3)},\,qQ,rs^{(3)}}$	77.3	63.1	80.8	58.9	1.05
$\sigma _{\varphi Q^ {(3)},\varphi Q^ {(3)}}$	0.434	0.354	0.485	0.354	1.12
$\sigma _{tW,tW}$	0.758	0.619	1.03	0.752	1.36
$\sigma _{itW,itW}$	0.761	0.616	1.03	0.752	1.35
$\sigma _{qQ,rs^{(3)},\,\varphi Q^ {(3)}}$	$-$ 5.49	$-$4.48	$-$ 6.08	$-$ 4.43	1.11
$\sigma _{qQ,rs^{(3)},\,tW}$	$-$ 2.34	$-$ 1.91	$-$ 2.84	$-$ 2.07	1.22
$\sigma _{\varphi Q^ {(3)},tW}$	0.182	0.148	0.212	0.155	1.17

**Table 2:** The benchmark choices for the coupling values of the effective operators, together with the corresponding *t*-channel single top cross section and the width of the top quark. The scale and PDF uncertainties of the cross sections are also shown

Operator	Coupling value	LO		NLO	
		$\sigma$[pb] ±scale ±PDF	$\Gamma _{\text {top}}$ [GeV]	$\sigma$[pb] ± scale ± PDF	$\Gamma _{\text {top}}$ [GeV]
$\text {SM}$	–	$123^{+9.3\text {\%}}_{-11.4\text {\%}} \pm 8.9\text {\%}$	1.49	$137^{+2.7\text {\%}}_{- 2.6\text {\%}} \pm {1.2\text {\%}}$	1.36
$O^{(3)}_{qQ,rs}$	$-$0.4	$172^{+8.7\text {\%}}_{- 10.8\text {\%}} \pm 8.9\text {\%}$	1.49	$190^{+2.4\text {\%}}_{- 1.8\text {\%}} \pm 1.1\text {\%}$	1.35
$O_{\varphi Q}^{(3)}$	1	$137^{+9.3\text {\%}}_{-11.4\text {\%}} \pm 8.9\text {\%}$	1.67	$154^{+2.3\text {\%}}_{- 2.3\text {\%}} \pm 1.2\text {\%}$	1.52
$O_{tW}$ (Re)	2	$132^{+9.3\text {\%}}_{-11.4\text {\%}} \pm 8.8\text {\%}$	1.83	$148^{+2.3\text {\%}}_{-2.5\text {\%}} \pm 1.2\text {\%}$	1.68
$O_{tW}$ (Im)	1.75i	$125^{+9.2\text {\%}}_{-11.4\text {\%}} \pm 8.8\text {\%}$	1.51	$140^{+2.3\text {\%}}_{- 2.5\text {\%}} \pm 1.2\text {\%}$	1.38

### Inclusive single top production

We start by computing the total single top production cross section for stable top quarks for the relevant operators at LO and NLO for the LHC, at 13 TeV. These results are also available in Ref. [6], but we reproduce them here in Table 1 for completeness. Our computation uses the five-flavour number scheme.^3^ For these results the renormalisation and factorisation scales, $\mu _{R}$ and $\mu _F$ are both set to $m_t = 172.5$ GeV. The NNPDF3.0 LO and NLO sets [27] are used for the LO and NLO predictions respectively and the only kinematic cuts are applied to the jets: $p_T^j > 5$ GeV and $|\eta _j| < 5$. To show the impact of the NLO corrections, Table 1 presents the *K*-factors which are defined as the ratio $\sigma _{\text {NLO}}/\sigma _{\text {LO}}$ for each contribution.

We find that for the single top process the squared terms and interference between the operators, i.e. the ${\mathcal {O}}(1/\Lambda ^4)$ terms, are suppressed for coefficients of ${\mathcal {O}}(1)$ for the $O_{tW}$ and $O_{\varphi Q^{3}}$ operators but are not negligible for the 4-fermion operator. Taking its coefficient to be of order one we find a large cancellation between the interference and squared contributions. We also observe that *K*-factors vary considerably between the various operators, and can be quite different from the SM contribution. This underlines the importance of including genuine NLO corrections in predictions, since a universal *K*-factor does not summarise the table. In the table we also include the contribution of the imaginary part of the coefficient $C_{tW}$, which only enters squared at ${\mathcal {O}}(1/\Lambda ^4)$ as it does not interfere with the SM or the other operators. We will discuss this contribution in detail in Sect. 3.5.

Total cross-section results give a good first indication on the impact of the operators on the single top production process, but more information can be extracted by considering differential distributions. To demonstrate the effect of the operators on the differential distributions we select a set of benchmark scenarios. The benchmark coupling values that will be used throughout the paper are presented in Table 2. We follow the EFT analyses of Refs. [6, 28] to ensure that our coupling values fall within the current limits. The effects on the inclusive cross section and the top width are also given for both LO and NLO. The predicted deviations from the SM predictions lie within the uncertainty of recent single top measurements: $\sigma = 156 \pm 35$ pb and $0.6 \le \Gamma _{\text {top}} \le 2.5$ GeV [29–32]. In the table we also include the scale uncertainties obtained by varying the central renormalisation and factorisation scale by a factor of two up and down, and the PDF uncertainties. We note the significant decrease in the scale and PDF uncertainties going from LO to NLO, a well-known feature of NLO computations. At NLO the combined uncertainty is only of the order of 3%, in agreement with previous results [22]. We shall therefore refrain from showing uncertainty bands in our differential distributions, even though these can be straightforwardly computed with our setup.

**Fig. 3:**
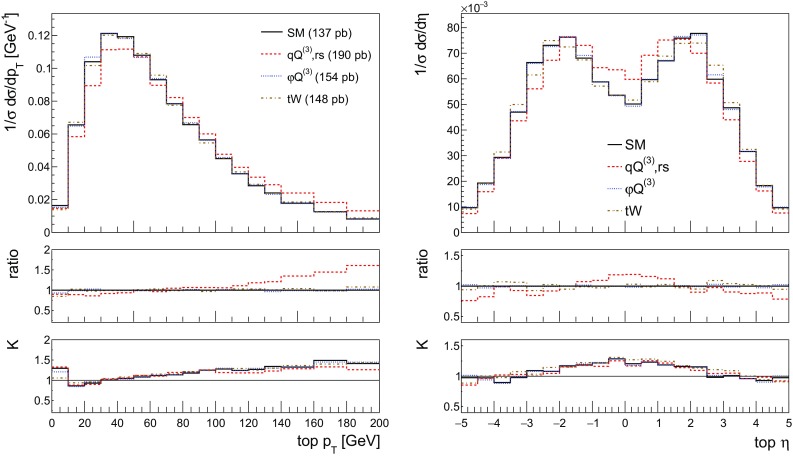
The NLO distributions of the stable top quark transverse momentum and rapidity for the SM and the three effective operators of interest Eqs. (1.2–1.4) for the couplings values of Table 2. The ratio shown in the first inset is defined as the effect of the operator over the SM, the second inset shows the *K*-factor, the ratio of the NLO over the LO predictions

We start by showing the stable^4^ top quark transverse momentum and pseudorapidity distributions for SM and the first three benchmarks of Table 2 in Fig. 3. Computing these distributions, we allow only one operator coefficient to be non-zero at a time. We include the interference with the SM as well as the square terms. In the distributions we do not include the benchmark with imaginary $C_{tW}$ coefficient which will be discussed in detail in Sect. 3.5.

In the distributions we see that the 4-fermion operator in particular has an effect on the shapes in both the transverse momentum and rapidity distributions, leading to harder and more central tops. The impact of the other two operators on the distribution shapes is milder. It can also be observed that the shape difference between LO and NLO has its largest effect in the sensitive region of these distributions, highlighting again the importance of NLO predictions for experimental analyses of this process.

### Single top production and decay

To study the process in more detail and extract maximal information on the impact of the operators, we should consider the distributions of the top decay products. This requires studying the full process of $p p \rightarrow b \ell \nu j$, where we have assumed that the top quark decays leptonically. In such a computation several difficulties arise compared to that for the inclusive $pp \rightarrow tj$ computation.

The first is that to generate a consistent single top event sample, the full process $p p \rightarrow b \ell \nu j$ has to be generated, including both the off-shell top effects and the interference with all the irreducible backgrounds. A NLO generation of the full process, though possible, is computationally too demanding for our purpose. We therefore adopt approximations involving the presence of either an intermediate top quark or a *W* boson. However, we should ensure that we do not lose any information about spin correlations. We thus generate the following samples.

- The full matrix element up to the leptons $(b\nu l j)$ in MG5_aMC (*fullchain*).
- *Wbj* production in MG5_aMC and decay the *W* in MadSpin (*halfchain*).
- Single top production (*tj*) in MG5_aMC and decay the top and *W* in MadSpin (*nochain*).

We have investigated the differences between the three methods at LO, where all are straightforward to implement. In particular, given that we wish to retain spin correlations in all three approaches, we examine the differences involving the polarisation angle $\theta _i^z$, the angle between the direction of decay product *i* and the spectator jet, as viewed in the top rest frame. The angular distribution of any top decay product in this frame can be parameterised as

3.4 $$\begin{aligned} \frac{1}{\sigma }\frac{d\sigma }{d \cos \theta ^z_i}=\frac{1}{2}\left( 1+a_i P\, \cos \theta ^z_i \right) , \end{aligned}$$

where *P* denotes the top quark polarisation and $a_i$ encodes how much spin information is transferred to each decay product. For the charged lepton $a_l$ is close to 1, indicating nearly 100% correlation.

**Fig. 4:**
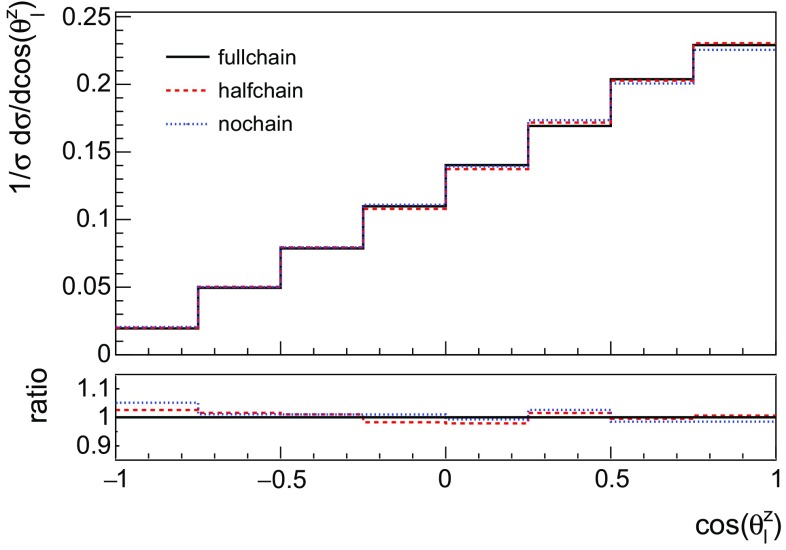
The SM top polarisation angle at LO for the 3 different generation options, as described in the text. The ratios with respect to the fullchain method are shown in the lower pane

All three options show good agreement, as shown in Fig. 4. We verified this to be the case for other observables as well. Given the level of agreement we find at LO between the *Wbj* and $l\nu b j$ distributions we will follow the *halfchain* method for our NLO results, i.e. we produce *Wbj* and decay the *W* in MadSpin, employing the relatively narrow *W*-width. A similar agreement is expected to hold at NLO, in particular as the leptonic decay of the *W* is not sensitive to higher order QCD corrections.

### Treatment of top quark width and impact of multiple operator insertions

In addition to the difficulties already present in the SM calculation for single top production and decay at NLO, the following EFT related subtleties affect the computation as well:

(i) The width of the top enters in the production of the *Wbj* final state. The effective operators affect the numerical value of this width, which has to be computed accordingly. We examine the modifications of the width value and its impact on the validity of the narrow width approximation for the top decay.
(ii) By considering the *Wbj* production matrix elements, the effective operators can now enter both in top production and in top decay. Allowing more insertions in the amplitude generates higher order terms in $1/\Lambda ^2$. These higher-order terms are expected to be suppressed but we will check this explicitly. Studying the *Wbj* final state moreover implies that configurations without top quarks contribute. The dimension-6 operators can affect also these irreducible backgrounds, hence their contributions should be included and their impact studied.

Let us address these two subtleties in turn.

(i)  As discussed in Eq. (3.2), the effect of one effective operator on the width of the top can be described by a second order polynomial $1/\Lambda ^2$, e.g. for $O_{tW}$ (real $C_{tW}$) the width takes the form:

3.5 $$\begin{aligned} \Gamma _{\text {top}}(C_{tW})= & {} \Gamma _{\text {SM}} + \frac{1\mathrm{TeV}^2}{\Lambda ^2} C_{tW} \, \Gamma _{tW} \nonumber \\&+ \frac{1\mathrm{TeV}^4}{\Lambda ^4} C^2_{tW} \, \Gamma _{tW,tW}. \end{aligned}$$

**Fig. 5:**
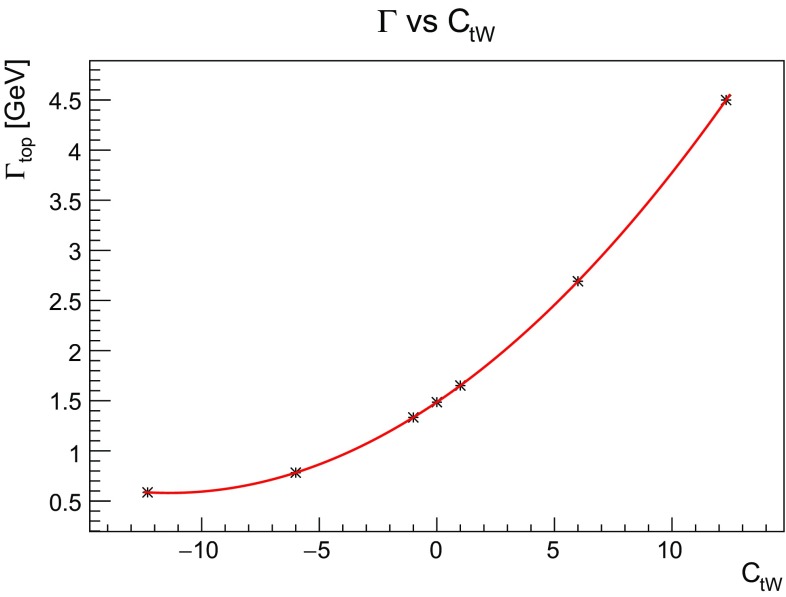
The top width as a function of the effective coupling $C_{tW}$ for $\Lambda =1$ TeV. The quadratic dependence on $C_{tW}$ is predicted by Eq. 3.5. At the indicated points the width is computed for the corresponding $C_{tW}$ values, while the line is a quadratic fit

In Fig. 5 we show how the top width, computed at LO, varies as a function of the operator coefficient $C_{tW}$, demonstrating the quadratic functional dependence. It is important to stress here that there are experimental constraints on the value of the width by both CMS and ATLAS [31, 32], as well as theoretical proposals [33] to extract more information about the top width.

When the width is small compared to the total mass of the particle, one can factorise the total cross section for a given decay channel into the production cross section multiplied by the branching ratio corresponding to that particular decay channel. This narrow width approximation (NWA) rests upon the following approximation for the denominator of the squared top quark propagator [34]:3.6The inclusive cross section of the single top production and decay to a *W* boson and a *b* quark is then approximated by:

3.7 $$\begin{aligned}&\sigma (pp \rightarrow Wbj) \rightarrow \sigma (pp \rightarrow tj) \, \frac{\Gamma (t \rightarrow Wb)}{\Gamma _{\text {top}}} \nonumber \\&\quad = \sigma (pp \rightarrow tj) \, \text {BR}(t \rightarrow Wb). \end{aligned}$$

**Fig. 6:**
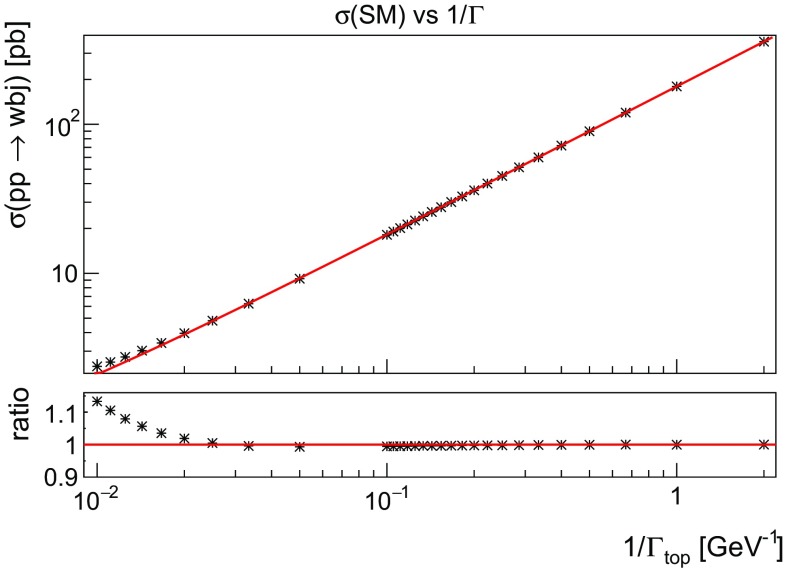
The SM cross section as a function of the width of the top. The NWA is valid when the relation is linear

**Fig. 7:**
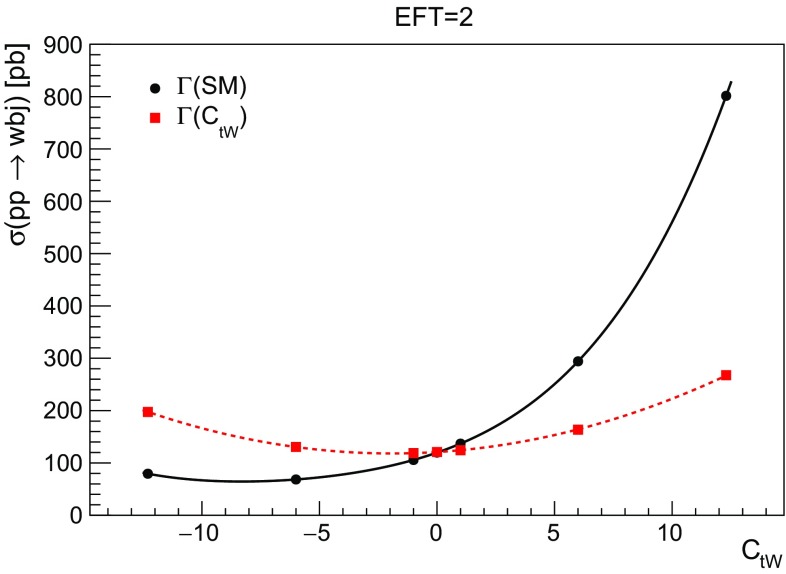
The cross section of the *Wbj* production as a function of $C_{tW}$ with two EFT insertions for the width of the top fixed to the SM value of 1.5 GeV (quartic dependence), or computed according to the value of the operator (quadratic dependence)

Since for top decays the branching ratio $\text {BR}(t \rightarrow Wb) \approx 1$, a direct way of testing the range of NWA validity in (3.7) is to calculate $\sigma (pp \rightarrow Wbj)$ at different numerical values of $\Gamma _{\text {top}}$, with SM couplings. This is shown in Fig. 6 where the linear dependence on $1/\Gamma _{\text {top}}$ can be observed for small $\Gamma _{\text {top}}$, whilst for $\Gamma _{\text {top}}> 50$ GeV the linear dependence breaks down. For non-excluded values of the operator coefficients the modifications of the width are moderate and therefore the NWA is expected to hold.

(ii) The same interactions occur, at leading order, in the production and the decay of the single top quark, hence the amplitudes for the process $\sigma (pp \rightarrow Wbj)$ can contain up to two insertions of an effective operator (to be precise of $O_{tW}$ or $O_{\varphi Q}^{(3)}$). The behaviour of the cross section as a function of the coefficient requires then a more complicated functional form than the one predicted by (3.3), in part due to the presence of more insertions, and in part due to the dependence of the top width on the coefficient, which enters in the *Wbj* calculation. The situation is however simplified in the NWA since the cross section for the production and the decay of a single top quark with two insertions of the effective coupling $C_{tW}$ can then be written schematically as:

3.8 $$\begin{aligned}&\sigma ^{pp \rightarrow Wbj}_{\text {EFT=2}}(C_{tW},\Gamma (C_{tW}))\nonumber \\&\quad \sim \left( \sigma _{\text {SM}} + C_{tW} \cdot \sigma _{tW} + C^2_{tW} \cdot \sigma _{tW,tW} \right) _{(\text {tj})}\,, \end{aligned}$$

where we have chosen $\Lambda = 1\, \mathrm {TeV}$ to avoid notational clutter. We shall also do this for Eq. (3.9) below. We have indicated by the subscript (*tj*) that the dependence of the partial *Wb* width and total width on $C_{tW}$ (and therefore in the branching fraction) cancels in Eq. (3.7). In other words, in the NWA the $C_{tW}$ dependence is as for producing a stable top quark plus jet. Figure 7 compares the case where the width is fixed to its SM value (1.5 GeV) with the case where the width is computed based on the coefficient value. In both cases two insertions are allowed in the amplitude. When working in the NWA, the width, being a function of the coefficient, eventually leads to a quadratic dependence of the cross section on $C_{tW}$ in (3.8). When one takes the width fixed there is no cancellation in the partial and total top width, and the dependence is quartic.

**Fig. 8:**
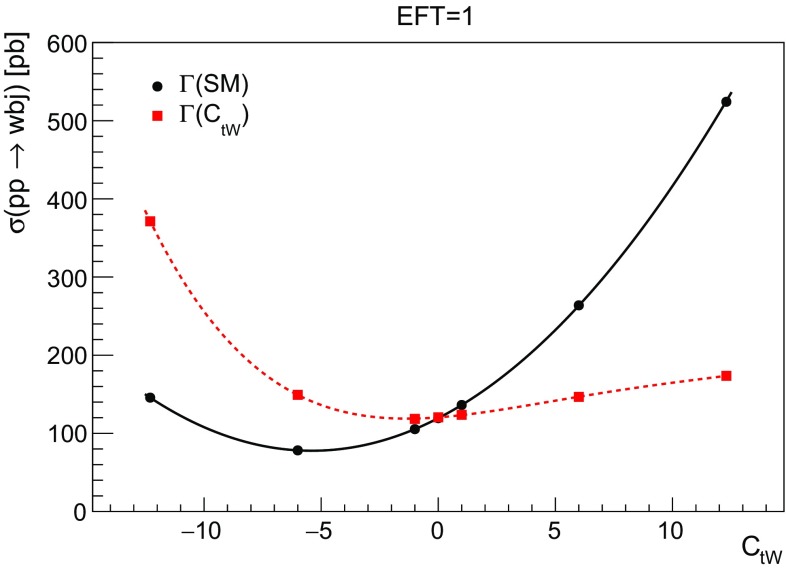
The cross section of *Wbj* production as a function of $C_{tW}$ with one insertion with the width of the top fixed to the SM value (quadratic behaviour) or computed according to the value of the operator (higher-order polynomial)

When only one insertion of an effective coupling is allowed (still in the NWA), it can enter either in the production or in the decay. The simplified form of the cross section in this case becomes:

3.9 $$\begin{aligned} \sigma ^{pp \rightarrow Wbj}_{\text {EFT=1}}(C_{tW}) \sim \frac{ \sigma _\text {SM} + C_{tW} \, \sigma _{tW} + C^2_{tW} \, \sigma _{tW,tW}}{\Gamma _{\text {SM}} + C_{tW} \, \Gamma _{tW} + C^2_{tW} \, \Gamma _{tW,tW}}, \end{aligned}$$

where $\sigma$ indicates that the *Wbj* final state is generated, with only one operator insertion. The $\Gamma$ in the denominator indicates that the cross section is described by the narrow width approximation. Since the terms in the numerator are different in their $1/\Lambda ^2$ dependence from the terms in the denominator, no cancellations occur. The impact of how the width is treated can be seen in Fig. 8 for the one-insertion calculations, where as expected a quadratic behaviour is observed when the width of the top is fixed, and a higher order polynomial is required to describe the behaviour when the width is computed with $C_{tW}$ dependence.

**Fig. 9:**
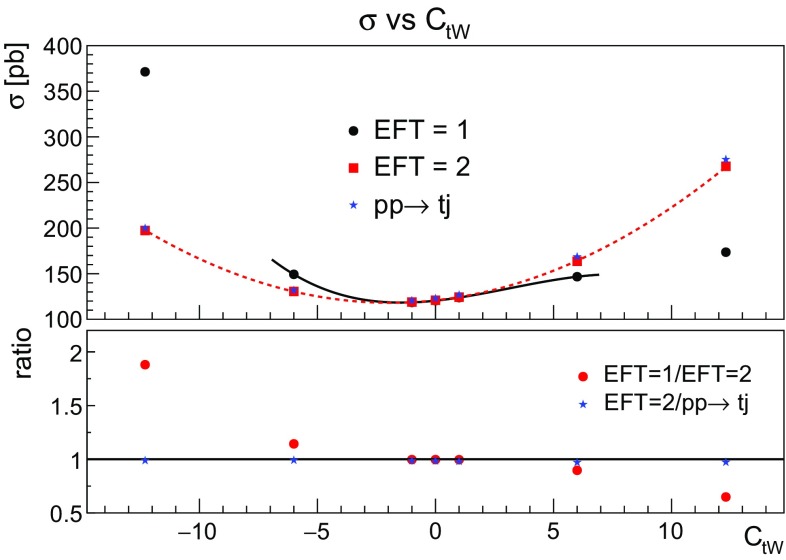
Comparing the different behaviour of the *Wbj* cross section with one $C_{tW}$ insertion (EFT = 1) or two $C_{tW}$ insertions (EFT= 2). Both effects have been discussed separately in Figs. 7 and 8. Additionally we show the production cross section ($pp \rightarrow tj$) which is reproduced by *Wbj* with two insertions when the right width is taken into account

Finally in Fig. 9 we compare the behaviour of the total cross section with one operator insertion (EFT=1) or two insertions (EFT = 2). It can be observed that for small values of the coupling, the linear term dominates and the cross sections coincide, as they only differ by higher order terms in $1/\Lambda ^2$. Notice that Fig. 9 also shows that the production cross section ($\sigma (pp \rightarrow tj)$) is very close to the *Wbj* cross section with two insertions of the couplings, as we expect from the NWA.

**Fig. 10:**
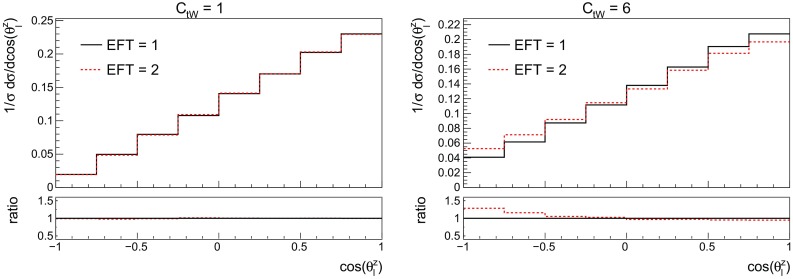
The top polarisation angle at LO with different values for the $O_{tW}$ effective operator. On the left hand side results for $C_{tW}=1$ are shown, whilst $C_{tW}=6$ on the right hand side. The comparison between one and two EFT insertions is shown

In order to examine whether the conclusions reached so far apply to differential distributions as well we show in Fig. 10 the top polarisation angle, defined in (3.4), obtained for two different values of the coefficient for one and two EFT insertions. The left pane shows both EFT options for $C_{tW}=1$. One can observe that the two distributions coincide within statistical errors. The right pane shows the case of $C_{tW}=6$, here the impact of higher order terms are important and these cannot be described by a global normalisation factor as shown in the ratio inset. This indicates that higher order effects in the EFT can be non-negligible. Therefore, for consistency with the production cross section and to avoid missing large higher order effects, all distributions in the rest of this paper have been obtained by generating *Wbj* allowing up to two EFT insertions, with the top width computed as a function of the coupling.

We note here that we validated our leading-order results with the ones discussed in [19] for the top-quark polarisation (*P*), analysing power ($a_i$) and lepton angular distributions. We performed a detailed comparison by allowing all possible insertions of the operators and matching all parameters of the computation with the one implemented in the generator Protos [18], and found perfect agreement.

### Results at NLO

Having studied the various effects at LO we proceed by computing the *Wbj* cross section at NLO in QCD in the presence of the dimension-6 operators. The *W* boson is decayed leptonically through MadSpin, and Pythia8 [35] is used for parton showering and hadronisation. Since we also generate the irreducible backgrounds, a loose invariant mass cut is imposed on the *Wb* system, centered on the top mass $100\, \mathrm {GeV}< M_{Wb-\mathrm {jet}} < 250\, \mathrm {GeV}$ [24]. Jet clustering is done using fastjet [36] and the anti-$k_t$ algorithm [37], with the jet radius parameter set to 0.4. All other generator settings and kinematic cuts are the same as in Sect. 3.1.

**Fig. 11:**
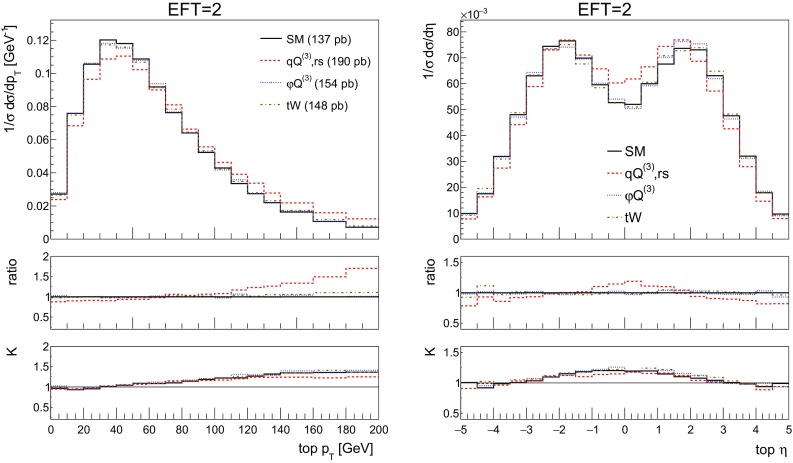
The NLO distributions of the reconstructed top transverse momentum (left) and rapidity (right) for the SM and the three effective operators of interest for the couplings values of Table 2. We quote in the left figure the corresponding inclusive cross section from this table. The ratio shown in the first inset is defined as the effect of the operator over the SM, the second inset shows the *K*-factor

We start by showing the top quark transverse momentum and rapidity distributions in Fig.  11 for the SM and the three operators, along with the ratio over the SM prediction and the corresponding *K*-factor. The top quark is now reconstructed from its semi-leptonic decay products, consisting of hardest electron, the associated neutrino and a *b*-jet. The light spectator jet is identified as well. When more than one *b*-jet is present we choose the one yielding the best reconstructed top mass. The results in Fig. 11 are in excellent agreement with those in Fig. 3.

**Fig. 12:**
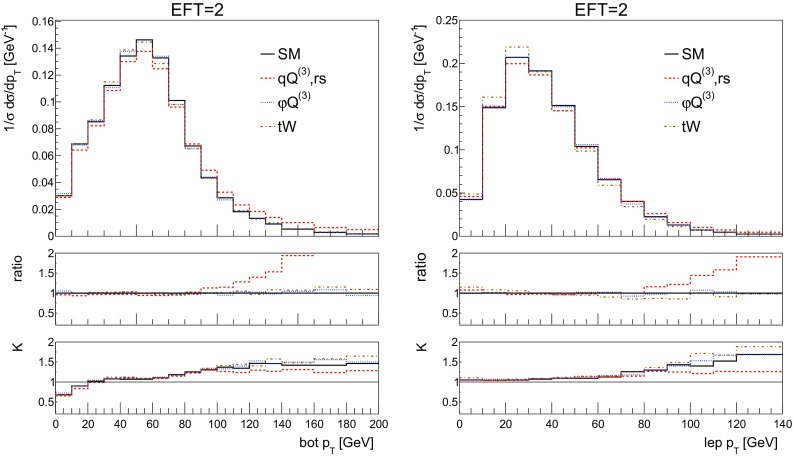
The NLO distributions of the $b-$jet (left) and electron (right) transverse momentum for the SM and the three effective operators of interest for the couplings values of Table 2. The ratio shown in the first inset is defined as the effect of the operator over the SM, the second inset shows the *K*-factor

Other observables of interest are the kinematic distributions of the lepton and *b*-jet from the decay of the top, shown in Fig. 12. Their $p_T$ distributions show a harder tail for the 4-fermion operator, whilst all contributions show a non-flat *K*-factor, with QCD corrections being larger in the high-$p_T$ region, for both the *b*-jet and the lepton.

Since the spin axis of the top is known [15] a rich set of angular observables showing spin correlations, can be exploited. Below we will elaborate on the definitions of the angles involved. In general, based on the choice of reference frame, it is possible to probe the production- and decay vertex of the single top separately. In any frame, a new set of coordinates can be defined based on the spin axis of the top. These additional coordinate axes provide the ability to construct other angles that contain spin information. For brevity, only the angular distributions that show the most sensitivity to the effective operators will be presented in this section.

The polarisation angle defined in Eq. (3.4) is one of the spin correlated angles that probes the production vertex. We use the same reference system as in [19] to construct a new set of coordinates:

3.10 $$\begin{aligned} {\hat{z}} = \frac{\vec {p_j}}{|\vec {p_j}|}, \;\;\;\;\;\; {\hat{y}} = \frac{\vec {p_j} \times \vec {p_q}}{|\vec {p_j} \times \vec {p_q}|}, \;\;\;\;\;\; {\hat{x}} = {\hat{y}} \times {\hat{z}}\,. \end{aligned}$$

The vectors $\vec {p}_j$ and $\vec {p}_q$ represent the direction of the spectator- and of the initial quark, respectively, both in the top quark rest-frame. Since the initial quark cannot be known with certainty, the beam axis is used.

We investigate the distributions of the angles between the directions of the top quark decay products and these new directions. The angle of the charged lepton with respect to the three axes defined above is affected most by the polarisation of the top [38].

**Fig. 13:**
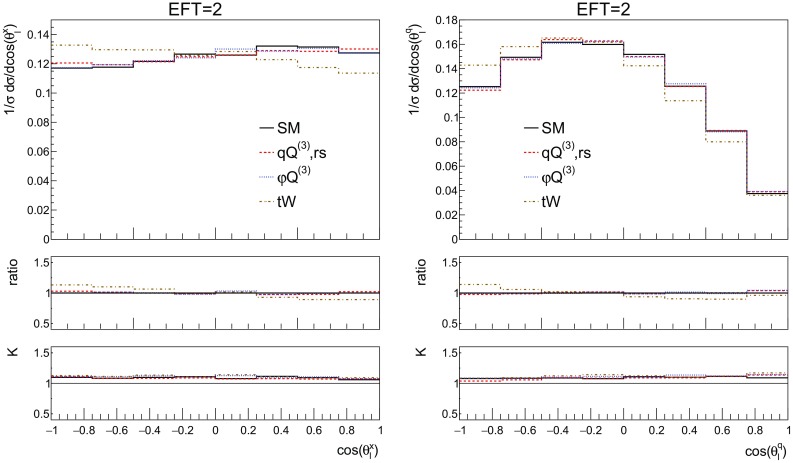
The NLO distributions of the top polarisation angle (left) and W helicity (right) for the SM and the three effective operators of interest for the couplings values of Table 2. The ratio shown in the first inset is defined as the effect of the operator over the SM, the second inset shows the *K*-factor

Figure 13 (left) shows the NLO distributions for $\cos \theta _l^x$, where $\theta _l^x$ is the angle between the lepton and direction ${\hat{x}}$. Notice that the dipole operator (${\mathcal {O}}_{tW}$) leads to a different distribution compared to the SM and the other operators.

**Fig. 14:**
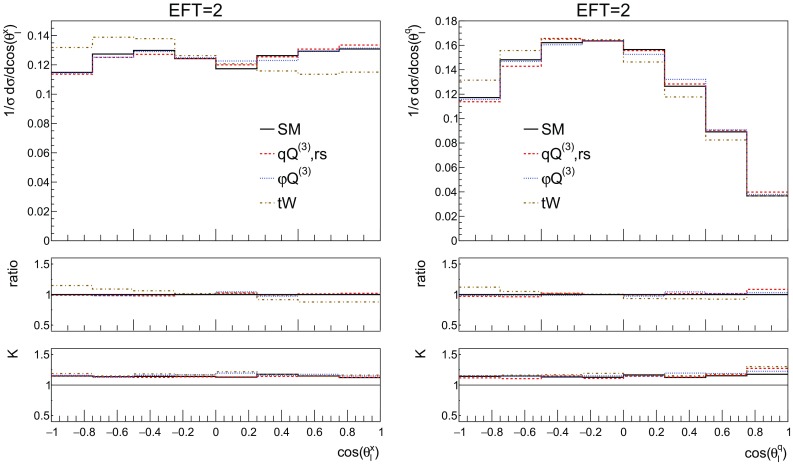
The NLO distributions of the top polarisation angle (left) and W helicity (right) for the SM and the three effective operators of interest for the couplings values of Table 2. The ratio shown in the first inset is defined as the effect of the operator over the SM, the second inset shows the *K*-factor. Here additional cuts are applied on the leptons: $p_T^l > 10$ GeV and $|\eta ^l| < 2.47$ and jets: $p_T^j > 20$ GeV and $|\eta ^j| < 4.5$

In order to probe new interactions in the decay of the top, one can examine the well-known $W-$helicity fractions $F_+, F_L$ and $F_0$ defined in:

3.11 $$\begin{aligned} \frac{1}{\Gamma } \frac{d\Gamma }{d \cos \theta _l^q}= & {} \frac{3}{8} \left( 1 + \cos \theta _l^q \right) ^2 F_+ + \frac{3}{8} \left( 1 - \cos \theta _l^q \right) ^2 F_- \nonumber \\&+ \frac{3}{4} \sin ^2 \theta _l^q F_0, \end{aligned}$$

where $\theta _l^q$ is the angle between the *W* in the top rest-frame and the charged lepton in the *W* rest-frame. $F_i$ represent the helicity fractions, with $\sum F_i = 1$.^5^ Again a new reference system can be constructed [40]:

3.12 $$\begin{aligned} {\hat{q}} = \frac{\vec {p_W}}{|\vec {p_W}|}, \quad {\hat{N}} = \frac{\vec {s_t} \times \vec {q}}{|\vec {s_t} \times \vec {q}|}, \quad {\hat{T}} = {\hat{q}} \times {\hat{N}}\,. \end{aligned}$$

The vectors $\vec {p}_W$ and $\vec {s}_t$ are both defined in the rest-frame of the top quark and depict the direction of the *W* boson and that of the top quark spin, respectively. The spin of the top quark is taken as the direction of the spectator jet [15, 16]. The angle of the lepton in the *W* rest-frame with respect to the three axes defined above probes the decay vertex. Figure 13 (right) shows the NLO distributions for cos $\theta _l^{q}$ where the sensitivity to the dipole interaction comes mainly in the $\theta _l^q\sim \pi$ region.

**Fig. 15:**
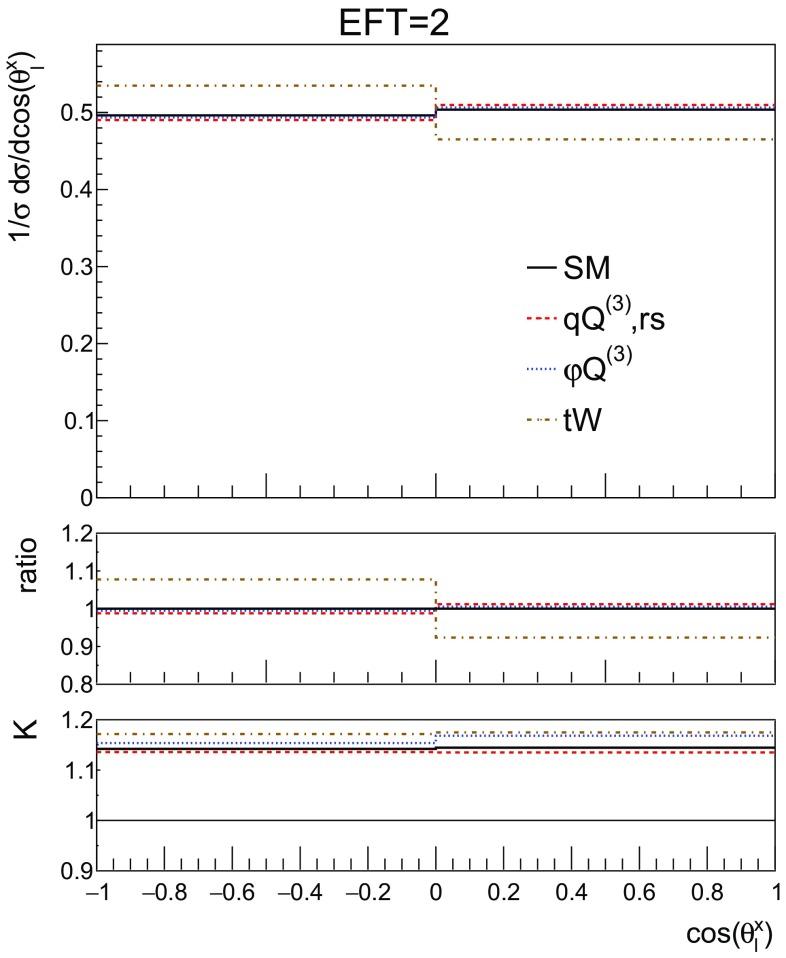
The asymmetry between positive and negative values of the top polarisation angle (cos $\theta _l^x$) by presenting Fig. 14 (left) in 2 bins

To show more realistic distributions, Fig. 14 shows the same observables as Fig. 13, only here additional cuts have been applied resembling the acceptance of the ATLAS detector. Namely, charged leptons must lie inside the $|\eta | < 2.47$ region and have a transverse momentum of at least 10 GeV, whereas jets should have a transverse momentum larger then 20 GeV and lie inside the $|\eta | < 4.5$ region. We note here that experimental selection cuts can potentially be more stringent in both rapidity, transverse momentum or angular separation observables of the different particles. Here we do not aim at reproducing the setup of the experimental analyses, but just to provide an indication of how selection cuts can affect the sensitivity to the dimension-6 effects. We find that our additional cuts lead to a significant reduction of the statistics and to a weakened sensitivity to the dimension-6 effects for the angular observables considered here. Despite the reduction in the sensitivity, the shape difference in the cos $\theta _l^x$ distribution (Fig.  14 left) between the dipole and other operators persists. This shape difference can be measurable as an asymmetry between positive and negative values of cos $\theta _l^x$ as can be seen in Fig. 15.

**Fig. 16:**
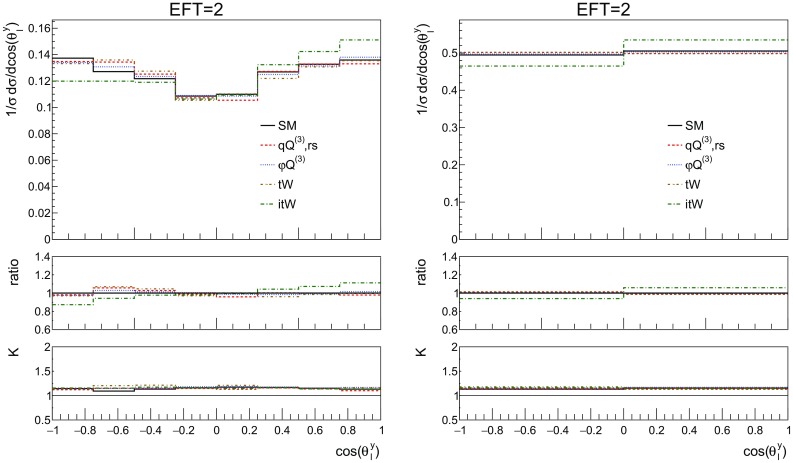
The NLO distributions of the top polarisation angle for the SM and the three effective operators of interest, together with the imaginary part of ${\mathcal {O}}_{tW}$ for the couplings values of Table 2. On the left the shape of the distribution can be seen, on the right the same distribution is shown in two bins where the asymmetry is clearly observed. The ratio shown in the first inset is defined as the effect of the operator over the SM, the second inset shows the *K*-factor. Here additional cuts are applied on the leptons: $p_T^l > 10$ GeV and $|\eta ^l| < 2.47$ and jets: $p_T^j > 20$ GeV and $|\eta ^j| < 4.5$

**Fig. 17:**
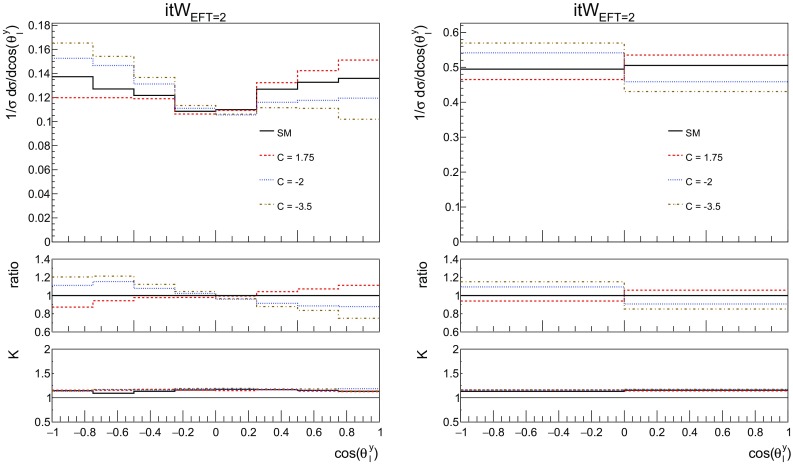
The NLO distributions of the top polarisation angle for the SM and different values for the imaginary part of ${\mathcal {O}}_{tW}$. On the left the shape of the distribution can be seen, on the right the same distribution is shown in two bins where the asymmetry is clearly observed. The ratio shown in the first inset is defined as the effect of the operator over the SM, the second inset shows the *K*-factor. Here additional cuts are applied on the leptons: $p_T^l > 10$ GeV and $|\eta ^l| < 2.47$ and jets: $p_T^j > 20$ GeV and $|\eta ^j| < 4.5$

We also mention here that we examined event samples where the operators were only allowed to enter in the production of the top quark. Here it was observed that for the *W* helicity angles, Eqs. 3.11 and 3.12, no deviation for the SM was observed. This validates that these angles probe the decay vertex only.

### CP-violation in single top

In this subsection we study possible CP-violating effects in single top production. In the SM CP violation is too small for baryogenesis, which motivates the search for new sources of CP-violation. Within the EFT, the coefficient of the ${\mathcal {O}}_{tW}$ operator can have an imaginary part, leading to a new CP-violating interaction. Here we study how large this effect could be and identify observables sensitive to it.

As discussed in [19], the polarisation angle $\cos {\theta ^y_{\ell }}$ defined in Eq. 3.10 shows a sensitivity to the phase of ${\mathcal {O}}_{tW}$ coefficient. This can indeed be observed in Fig. 16, where an asymmetry is clearly visible, for the imaginary part of the coefficient. The SM, charged current, four-fermion operator and real part of the dipole operator show no asymmetry in this distribution.

In order to focus on the effects of the imaginary part of $C_{tW}$, Fig. 17 shows results for a range of coupling values that are within the current global limits [28]. It is interesting to see that this observable is sensitive to both the size and to the sign of the coupling for $\mathrm {Im}\,{\mathcal {O}}_{tW}$. We note here that we additionally studied the asymmetry suggested in [3], but found this to be less sensitive to $\text {Im}C_{tW}$ than $\cos {\theta ^y_{\ell }}$.

## Conclusions

Single top production provides an excellent opportunity of probing top quark couplings. The SMEFT is a framework which allows us to parametrise deviations from the SM couplings in a consistent and model-independent way. Predictions in the SMEFT can be systematically improved by computing higher-order corrections. In this work we computed for the first time single top production and decay at NLO in QCD, in the presence of dimension-6 operators.

We studied the impact of these QCD corrections, both at the inclusive and differential level, and found that NLO effects affect both the total rates and the differential distributions in a non-trivial way, with different operator contributions receiving different *K*-factors. NLO effects can be large and are therefore needed to reliably predict the impact of the dimension-6 operators. We computed all relevant contributions at ${\mathcal {O}}(1/\Lambda ^2)$ (and some ${\mathcal {O}}(1/\Lambda ^4)$ terms), and examined their relative importance.

We then included also the decay of the top, examining the validity of the NWA and the impact of the top width in computing results for the *Wbj* final state. We find that the impact of the dimension-6 operators on the top width needs to be taken correctly into account to ensure that the *Wbj* and *tj* cross sections are consistent. We then computed top production and decay at NLO matched to the parton shower using the resonance-aware matching within MG5_aMC, including off-shell and interference effects. We obtained NLO distributions for both the top and its decay products for the SM and a series of benchmarks with non-zero operator coefficients. We find that the weak dipole and four-fermion operators can lead to harder tails in the distributions.

In order to fully exploit the power of spin correlations, we explored a series of angular observables that can be used to probe new physics couplings in either the production or decay of the top. These include the so-called polarisation angle and *W* helicity fractions. We find these angular distributions to be sensitive to different operators. The sensitivity becomes weaker when we apply cuts on the top decay products, but can still be probed by defining the corresponding asymmetries. Finally we considered CP-violating effects coming from the imaginary part of the dipole operator coefficient and studied an angular distribution that can be used to identify such an interaction.

Our study is an example of using an accurate and realistic simulation framework to compute deviations from the SM within SMEFT for a limited number of operators. Our results can be used in combination with the experimental results to obtain reliable constraints on the operator coefficients as part of the on-going effort of EFT interpretations of LHC top-quark measurements [41].

## Footnotes

1 Only electronic *W* decays are used in this study. Similar results are expected for top quark events with a muon in the final state. Events where the *W* is decaying to a $\tau$, or hadronically, are experimentally more difficult to isolate.

2 We remark that a slightly different approach [17–21], not using operators but anomalous couplings, has also been used in the literature. The connection between the operator coefficients to the anomalous couplings is discussed in [4]. Here all types of Lorentz-invariant interaction structures that involve the *W* boson and the top quark are allowed, including those that the Standard Model does not allow. An advantage of the present approach is the limited number of parameters, a restriction following from symmetry requirements.

3 We note here that a comparison between the four-flavour and five-flavour number schemes for the SM *t*-channel single top production can be found in the literature [25, 26]. Differences between the two schemes are reduced at NLO, but can be up to 10% at the LHC, depending on the observable. A detailed comparison of the two schemes is beyond the scope of this work, but we expect our conclusions regarding the distribution shapes and *K*-factors of the dim-6 contributions in comparison to the SM to hold also in the four-flavour scheme.

4 This top is selected based on its particle ID (i.e. in this example it is not reconstructed from its decay products), and therefore stable. We note that this is the case only for Sect. 3.1. In the following sections of the paper, the top is reconstructed from its decay products.

5 For a fit of *Wtb* anomalous couplings using the *W*-helicity fractions and single top cross-section measurements, see Ref. [39].
